# Association between Metformin Use and Risk of Total Knee Arthroplasty and Degree of Knee Pain in Knee Osteoarthritis Patients with Diabetes and/or Obesity: A Retrospective Study

**DOI:** 10.3390/jcm11164796

**Published:** 2022-08-17

**Authors:** Shibo Chen, Guangfeng Ruan, Muhui Zeng, Tianyu Chen, Peihua Cao, Yan Zhang, Jia Li, Xiaoshuai Wang, Shengfa Li, Su’an Tang, Shilong Lu, Tianxiang Fan, Yang Li, Weiyu Han, Jianye Tan, Changhai Ding, Zhaohua Zhu

**Affiliations:** 1 Clinical Research Centre, Zhujiang Hospital, Southern Medical University, Guangzhou 510280, China; 2 Department of Orthopaedics, Zhujiang Hospital, Southern Medical University, Guangzhou 510280, China; 3 Clinical Research Centre, Guangzhou First People’s Hospital, Guangzhou 510180, China; 4 Division of Orthopaedic Surgery, Department of Orthopaedics, Nanfang Hospital, Southern Medical University, Guangzhou 510515, China; 5 Department of Radiology, Zhujiang Hospital, Southern Medical University, Guangzhou 510280, China; 6 Menzies Institute for Medical Research, University of Tasmania, Hobart, TAS 7000, Australia

**Keywords:** osteoarthritis, metformin, total knee arthroplasty, knee pain, propensity score weighting

## Abstract

Objectives: We aimed to examine whether metformin (MET) use is associated with a reduced risk of total knee arthroplasty (TKA) and low severity of knee pain in patients with knee osteoarthritis (OA) and diabetes and/or obesity. Methods: Participants diagnosed with knee OA and diabetes and/or obesity from June 2000 to July 2019 were selected from the information system of a local hospital. Regular MET users were defined as those with recorded prescriptions of MET or self-reported regular MET use for at least 6 months. TKA information was extracted from patients’ surgical records. Knee pain was assessed using the numeric rating scale. Log-binomial regression, linear regression, and propensity score weighting (PSW) were performed for statistical analyses. Results: A total of 862 participants were included in the analyses. After excluding missing data, there were 346 MET non-users and 362 MET users. MET use was significantly associated with a reduced risk of TKA (prevalence ratio: 0.26, 95% CI: 0.15 to 0.45, *p* < 0.001), after adjustment for age, gender, body mass index, various analgesics, and insurance status. MET use was significantly associated with a reduced degree of knee pain after being adjusted for the above covariates (β: −0.48, 95% CI: −0.91 to −0.05, *p* = 0.029). There was a significantly accumulative effect of MET use on the reduced risk of TKA. Conclusion: MET can be a potential therapeutic option for OA. Further clinical trials are needed to determine if MET can reduce the risk of TKA and the severity of knee pain in metabolic-associated OA patients.

## 1. Introduction

Osteoarthritis (OA) is a chronic musculoskeletal disorder and one of the major causes of disability among the elderly. In China, the prevalence of symptomatic OA is 8.1% and is expected to increase due to accelerating aging [1]. Currently, there are no effective drugs to attenuate OA progression [2,3]. In the advanced disease stages of knee OA, total knee arthroplasty (TKA) is often required to relieve pain and improve function, though the patients may suffer from postoperative complications, especially over-aged patients [4]. Therefore, it is urgent to find effective drugs to control the symptoms and delay the progression of knee OA.

OA is a heterogeneous disease caused by multiple factors [5]. Studies have shown that the recognition of different phenotypes in patients with knee OA is highly correlated with the therapeutic effects of the disease [6]. Therefore, precise treatments targeting different OA phenotypes have emerged as promising therapeutic strategies. Metabolic syndrome-associated OA (MetS-OA) is a prevalent phenotype of OA [7]. Thus far, the mechanism underlying the association between MetS and OA is still unclear. The mainstream view is that the local effects of oxidative stress and low-grade systemic inflammation may exacerbate OA progression [8].

Metformin (MET) is a first-line drug for type 2 diabetes [9]. In addition to lowering blood glucose, MET has been proved to have weight loss, anti-inflammatory, and chondroprotective effects [9,10]. It has also been demonstrated that OA rats receiving MET treatment had a better pain tolerance, suggesting that MET may attenuate OA development and progression [11]. Furthermore, some basic research studies have reported that MET may alleviate OA via attenuating osteoclast-mediated abnormal subchondral bone remodeling, reducing chondrocyte pyroptosis, and inhibiting chondrocyte ferroptosis [12,13,14]. In addition, these pleiotropic effects of MET have been described to occur mainly through activating the adenosine monophosphate-activated protein kinase (AMPK) pathway [11].

Some observational studies have demonstrated a protective effect of MET against OA. A population-based cohort study showed that the combination of MET and COX-2 inhibitors can reduce the incidence of TKA in patients with OA and type 2 diabetes [15]. Another cohort study exploring the association between MET and OA found that the annual loss of medial cartilage volume in MET users was significantly lower than that in non-users [16]. However, a study in the United Kingdom showed no correlation between MET prescription and incidence of OA [17]. A systematic review of pre-clinical and human studies of the effects of MET on OA concluded that MET exerted favorable effects on chondroprotection, immunomodulation, and pain reduction in knee OA. Notably, there was only one study detecting the association between MET use and the severity of knee pain, which is considered the most important symptom of OA [18].

MET may play a potential therapeutic role in targeting MetS-OA by regulating inflammatory and metabolic factors. However, clinical studies evaluating the effect of MET on MetS-OA are rare and inconclusive. Therefore, the purpose of this study was to examine whether MET use is associated with a reduced risk of TKA and decreased knee pain severity in knee OA patients with diabetes and/or obesity.

## 2. Materials and Methods

### 2.1. Participants

**Figure 1:**
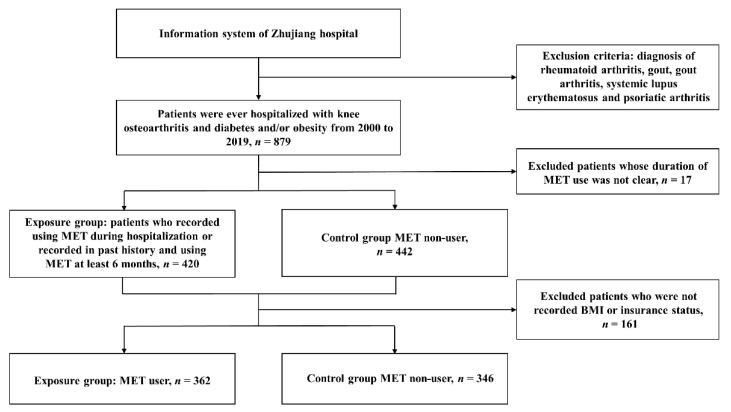
The flowchart of study population selection. Missing data had been excluded before performing IPTW.

This was a retrospective, cohort study using the routinely recorded information from a local hospital. Inclusion criteria were as follows: participants aged more than 18 years old; hospitalized in the local hospital from 10 June 2000 to 15 July 2019; discharge diagnosis was “knee osteoarthritis” or “knee osteoarthropathy” or “degeneration of the knee joint” combined with “diabetes” and/or “obesity”. Patients with inflammatory arthritis including gout, reactive arthritis, rheumatoid arthritis, psoriatic arthritis, and systemic lupus erythematosus were excluded (Figure 1). We collected the anonymized data of participants from the hospital information system using “Data Process and Application Platform” (DPAP), developed by Yidu Cloud (https://www.yiducloud.com.cn/) (accessed on 12 December 2019). The study was approved by the institutional review board and ethics committee at a local hospital, and informed consent was waived.

### 2.2. Exposure

Participants were classified as regular MET users if they were recorded using MET following the doctor’s advice or self-reported regular MET use in past medical history and had been using MET for at least 6 months. Non-users of MET were defined as participants who were not recorded using MET at physician orders or did not ever report the use of MET or whose MET was first administered after TKA or who used MET for less than 6 months. Additionally, we further calculated the duration of MET use based on the sum of each duration of MET use and classified it as short-term and long-term MET use, using the median (2100 days) as the cutoff point. Patients who recorded only one time for using MET or did not record the time of use in their medical history were excluded from the classification of the duration of MET. In addition, the total MET dose was calculated based on the sum of each MET single dose multiplied by the duration of each MET use. We further classified the total MET dose as low dose and high dose, using the median (1048 g) as the cutoff point.

### 2.3. Outcome Assessment

#### 2.3.1. Total Knee Arthroplasty

In this scheme, TKA was regarded as the end point of OA. “Knee arthroplasty” was included in the surgical name screened from the database as the occurrence of TKA. Uni-compartmental knee arthroplasty (UKA) was excluded.

#### 2.3.2. Knee Pain

Knee pain was assessed using the numeric rating scale (NRS) ranging from 0 to 10 [19]. The degree of knee pain was collected from the medical record at admission, which was only evaluated in the resting state, with 0 representing no pain and 10 representing the most intensive pain. To minimize information bias, we excluded individuals whose medical records included hemiplegia and acute knee injury, which can impact the assessment of knee pain.

### 2.4. Statistical Analysis

The propensity score (PS) combining the covariates including age, sex, body mass index (BMI), insurance status, and the use of celecoxib, etoricoxib, and tramadol was weighted using the inverse probability of treatment weighting (IPTW) method to balance the covariates between MET user and non-user groups as well as the group using MET more than one year and the group using MET less than one year [20]. Specifically, MET users were assigned a weight equal to the reciprocal of the propensity score (1/PS), while MET non-users were assigned a weight equal to the reciprocal of one minus the propensity score (1/1-PS). The distribution of PS between the two groups basically overlapped, and there were few proportions of extreme values in the PS model (Figure S1 in the Supplementary Information). After propensity score weighting (PSW) using the IPTW method, the balance performance of covariates between the two groups was diagnosed using absolute standardized differences (ASDs). Generally, ASDs < 0.1 suggest that the differences between the two groups are negligible [21]. Missing data were excluded before the IPTW method and multivariable regression analyses were conducted. Log-binomial regression analyses were performed to obtain the prevalence ratio (PR) and 95% confidence intervals (CIs) of the association between MET use and TKA risk before and after adjustment for all the above covariates. Regarding the association between MET use and knee pain, linear regression analyses were performed before and after adjustment for all the above covariates.

A post hoc power calculation showed that we had more than 80% statistical power to detect a correlation between MET prescription and TKA risk, with an alpha error of 0.05 and two-sided significance.

Statistical analyses were conducted using Stata/MP 14.0 for Windows (StataCorp, College Station, TX, USA) and R software (3.6.3; R Foundation for Statistical Computing, Vienna, Austria). *p* value < 0.05 (double-tailed) or 95% confidence interval (CI) for β not including 0 (linear regression) or 95% CI for PR not including 1 (log-binomial regression) were considered statistically significant.

## 3. Results

### 3.1. Characteristics of Participants

**Figure 2:**
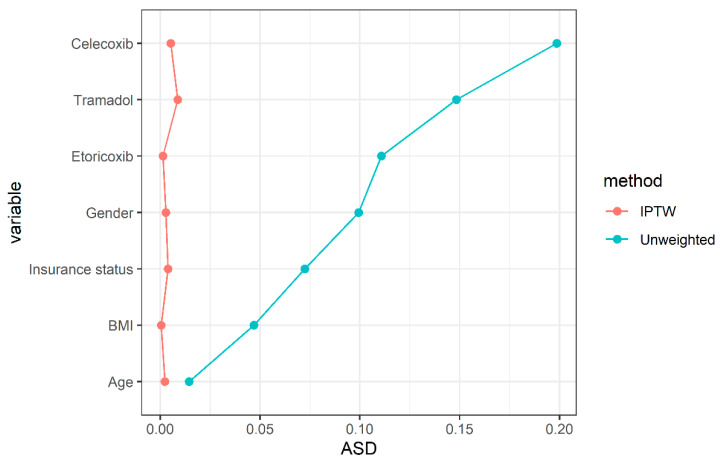
Distribution of absolute standardized differences in the unweighted and weighted samples.

The characteristics of the participants are shown in Table 1. Among the 862 participants included in the present study, 420 (48.7%) were MET users, and 442 (51.3%) were MET non-users. Before IPTW, MET users had higher rates of celecoxib and etoricoxib usage and lower rates of tramadol usage than non-users. There were no significant differences in age, gender, BMI, and insurance status between the two groups. After excluding patients who had missing data on BMI and insurance status, there were 362 MET users versus 346 MET non-users. Then, we performed the IPTW method to attenuate the imbalance between the two groups. After IPTW, common analgesics including celecoxib, etoricoxib and tramadol were well-balanced between the groups (all ASDs < 0.01) (Table 1 and Figure 2).

### 3.2. Associations between MET Use and Risk of TKA and Degree of Knee Pain

Table 2 illustrates the results of associations between MET use and the risk of TKA and the degree of knee pain before and after IPTW.

Before IPTW, 5% of the patients in the MET-user group performed TKA, while 19% of the patients performed TKA in the MET-non-user group. The MET users had an average knee pain score of 1.6, while MET non-users had an average knee pain score of 2.1. MET use was significantly associated with a decreased risk of TKA before and after adjustment for age, gender, BMI, insurance status, and the use of celecoxib, etoricoxib, and tramadol (*p* < 0.001, *p* < 0.001, respectively). MET use was significantly associated with a lower degree of knee pain before and after adjustment for the above covariates (*p* = 0.008 and *p* = 0.029, respectively).

After IPTW, 6% of the patients in the MET-user group performed TKA, while 21% of the patients performed TKA in the MET-non-user group. The average knee pain scores were the same as the ones before IPTW. MET use was still significantly associated with a reduced risk of TKA (*p* < 0.001). Moreover, MET use was still significantly associated with a lower degree of knee pain (*p* = 0.031).

### 3.3. The Accumulative Effect of MET Use on the Risk of TKA and Severity of Knee Pain

Compared with MET non-use, the risk of TKA decreased significantly among short-term and long-term MET use, respectively, in univariable and multivariable models (*p* < 0.001, *p* < 0.001, *p* = 0.003 and *p* < 0.001, respectively). Additionally, there were significant differences in TKA risk between short-term MET use and long-term MET use. After IPTW, the findings were largely similar. Taken together, there was a significant accumulative effect of MET use on the reduced risk of TKA (all *p* for trend <0.001) (Table 3).

Regarding the severity of knee pain, short-term MET use was significantly associated with a lower degree of knee pain in both univariable and multivariable models (*p* = 0.020 and 0.042, respectively), compared with MET non-use. Although long-term MET use was not significantly associated with knee pain, compared with MET non-use, there was a trend of an association between long-term MET use and the degree of knee pain (*p* = 0.065 and 0.164, respectively). After IPTW, the results were largely similar. Taken together, the increased duration of MET use was significantly associated with the reduced severity of knee pain before but not after adjusting for covariates (*p* for trend = 0.017 and 0.062, respectively) (Table 4).

### 3.4. The Dose-Dependent Effect of MET Use on the Risk of TKA and Severity of Knee Pain

Compared with MET non-users, the risk of TKA decreased significantly among those using low doses of MET and high doses of MET, respectively (both *p* for trend <0.001). After IPTW, the findings were largely similar. Taken together, there was a significant dose-dependent effect of MET use on the reduced risk of TKA (Supplementary Table S1).

Regarding the severity of knee pain, compared with MET non-users, those who used low doses but not high doses of MET were significantly associated with having lower severity of knee pain in both univariable and multivariable models (*p* = 0.014, *p* = 0.131, *p* = 0.025 and *p* = 0.269, respectively). After IPTW, the results were largely similar. Taken together, there was no significant dose-dependent effect of MET on the reduced severity of knee pain (*p* for trend = 0.024 and 0.094, respectively) (Supplementary Table S2).

## 4. Discussion

Using the data collected from the information system of a local hospital, the current study found that MET use was significantly associated with a decreased TKA rate and knee pain severity, compared with MET non-use, among patients with OA combined with diabetes and/or obesity. Meanwhile, increased duration of MET use was significantly associated with a reduced risk of TKA, suggesting a potential accumulative effect of MET use on the reduced risk of TKA. An increased total MET dose was significantly associated with a reduced risk of TKA, indicating that there may be a potential dose-dependent effect of MET use on the risk of TKA.

Recent studies found that, besides the hypoglycemic effect, MET may attenuate OA through alleviating inflammation, protecting cartilages, and reducing pain [10,22]. Nevertheless, there are very few clinical research studies examining the associations between the use of MET and OA outcomes, and the results were inconsistent. In a retrospective cohort study of patients with OA and type 2 diabetes, MET combined with COX-2 inhibitors reduced the risk of joint replacement surgery compared with COX-2 inhibitors alone [15]. Another prospective cohort study using Osteoarthritis Initiative (OAI) participants with knee OA and obesity revealed that MET use was associated with a reduced rate of medial knee cartilage volume loss over four years and with a trend toward a significant reduction in the risk of total knee replacement over six years [16]. By contrast, a retrospective cohort study with up to 10 years of follow-up found no significant association between the prescription of MET and diagnosis of OA in diabetes patients [17]. Consistent with previous studies, our study found solid evidence to support a significant association between MET use and TKA occurrence and the degree of knee pain before and after the adjustment of clinically important covariates. Moreover, our study supplemented the analyses of accumulative and dose-dependent effects of MET use with the reduced risk of TKA as well as the degree of knee pain, which, to some extent, fills the gap in previous studies [18].

In terms of knee pain, considering the mild knee pain in the OAI cohort, Wang et al. did not find a significant association between MET use and change in WOMAC pain over four years [16]. Collecting knee pain information in hospitalized patients, the current study showed a reduction in knee pain in MET users, compared with non-users. However, there were no significant accumulative and dose-dependent effects of MET use on the degree of knee pain. This may be explained by the fact that the minimum duration of MET use was defined as 6 months in our study. A previous randomized clinical trial found that increased duration of MET use was associated with a reduced pain score using the Knee Injury and Osteoarthritis Outcome Score (KOOS) within 12 weeks [23]. Thus, further large-sample size prospective cohorts and clinical trials are needed to confirm the causal association of accumulative MET use with the degree of knee pain.

The biological mechanisms linking MET to OA are largely unclear. Previous studies revealed that MET could modulate and weaken pro-inflammatory responses induced by lipopolysaccharide (LPS) in monocytes and macrophages [24]. These anti-inflammatory properties of MET were exerted irrespective of diabetes mellitus status [25]. Another experimental study showed that MET can enhance the anti-inflammatory and chondroprotective effects of mesenchymal stem cells [10], suggesting that MET may play anti-inflammatory and chondroprotective roles to delay the progression of OA [11]. Furthermore, previous studies have indicated that MET may attenuate neuropathic pain, and this effect may be through the activation of opioidergic mechanisms or regulation of pain mediators via the autophagy-lysosomal pathway [26,27,28]. In terms of the molecular pathways of MET effects on OA, most previous studies described that AMPK activation can modulate the chondroprotective, immunomodulatory, and analgesic effects of MET [18]. In addition, SIRT3/PINK1/Parkin activation and the downregulation of the mTORC1 pathway were also the potential molecular pathways of MET effects on OA [18].

The current study may have some clinical implications. At present, there is still a lack of effective drugs for the treatment of OA. Clinically, as the primary drugs for controlling OA symptoms, the long-term use of NSAIDs will not reduce or even increase the risk of TKA [29,30,31]. Moreover, there are many contraindications to the use of NSAIDs for the control of chronic pain, such as gastrointestinal comorbidities and cardiovascular comorbidities, which coincidentally occur in the elderly [32,33,34]. Notably, a network meta-analysis reported that there was uncertainty about the change in pain in knee OA patients for the long-term use of NSAIDs [35]. As a traditional hypoglycemic drug, MET has been shown to have anti-inflammatory effects [10]. Furthermore, various research studies have revealed that, as a hypoglycemic drug with few side effects, MET can be considered a potential compound for adjuvant therapy in bone disorders including OA [9,36]. In the meantime, an increasing amount of evidence suggests that MET may attenuate chronic pain and the progression of OA in animal experiments [27,28,37]. In general, if replicated and determined to likely be causal, our findings indicated that MET can be a potential therapeutic option for OA disease-modifying and symptom relief. In addition, our findings provide evidence for subsequent prospective cohort studies and clinical trials.

Regarding the association of MET and the progression of knee OA in patients without diabetes mellitus, the current study did not have sufficient samples to assess this association (data not shown). A previous study investigating the association between MET use and disease progression in obese people with knee OA found that MET use was significantly associated with lower rates of medial cartilage volume loss, indicating that MET may have protective effects on knee OA progression in people without diabetes mellitus [16]. Moreover, basic scientific studies focusing on the effects of MET on experimental OA mice models also indicated that MET may have a protective effect on OA in non-diabetic subjects [11,13,22].

The current study is the first to demonstrate an association between MET use and a lower risk TKA and lower severity of knee pain in osteoarthritic patients combined with diabetes and/or obesity. However, this study has several potential limitations. First, subject to the hospital information system, this study used a cross-sectional design. Second, patients with a prescription of MET had a higher rate of COX-2 inhibitors usage, a lower rate of tramadol usage, and a lower degree of knee pain before PSW. Therefore, while PSW was used to control potential confounding effects, residual confounding factors still could have affected the findings of this study. Third, the current study was conducted among OA patients with diabetes and/or obesity. Hence, these findings may not be generalizable to all OA patients. Fourth, the current study lacks relative data on the side effects of MET; it should be considered that some patients may experience side effects such as gastrointestinal side effects (e.g., diarrhea, nausea) and B12 deficiency. Fifth, due to a lack of radiographic OA data, we could not examine the association between MET use and the progression of radiographic OA.

## 5. Conclusions

MET can be a potential therapeutic option for OA. Further clinical trials are needed to determine if MET can reduce the risk of TKA and the severity of knee pain in metabolic-associated OA patients.

## Figures and Tables

**Table 1:** Characteristic between metformin users and non-users before and after IPTW using propensity score method.

	Before IPTW			After IPTW		
	Non-MET (*n* = 442)	MET (*n* = 420)	ASD ^‡^	Non-MET (*n* = 346)	MET (*n* = 362)	ASD
Age	73.8 ± 11.0	73.6 ± 10.3	0.014	72.7 ± 10.7	72.7 ± 10.1	0.002
Female	79%	73%	0.099	77%	77%	0.003
BMI, kg/m^2^	25.87 ± 4.3	25.9 ± 4.2	0.027	25.8 ± 4.4	25.8 ± 4.3	<0.001
Rural cooperative medical care system ^†^	79%	82%	0.072	82%	82%	0.004
Celecoxib	51%	61%	**0.199**	58%	58%	0.005
Etoricoxib	20%	25%	**0.111**	24%	24%	0.001
Tramadol	16%	11%	**0.148**	14%	13%	0.009

Data presented as mean (standard deviation) or proportion (%). Abbreviations: BMI, body mass index; MET, metformin; IPTW, inverse probability of treatment weighting; ^†^ insurance status can be categorized into the rural cooperative medical care system and medical services at state expense; ^‡^ ASD, absolute standardize difference, ASD < 0.1 indicates an ignorable difference. ASD values greater than 0.1 are shown in bold.

**Table 2:** Associations between metformin use and the risk of total knee arthroplasty and degree of knee pain.

	No. (Rate %) of TKA		PR (95% CI)	Mean ± SD of Knee Pain		β (95% CI)
	MET Non-Users	MET Users		MET Non-Users	MET Users	
Univariable ^†^	83 (19%)	21 (5%)	**0.23 (0.13, 0.37)**	2.1 ± 1.7	1.6 ± 1.9	**−0.54 (−0.93, −0.14)**
Multivariable ^‡^	72 (21%)	20 (6%)	**0.26 (0.15, 0.45)**	2.1 ± 1.7	1.6 ± 1.9	**−0.48 (−0.91, −0.05)**
IPTW ^§^	72 (21%)	20 (6%)	**0.29 (0.17, 0.49)**	2.1 ± 1.7	1.6 ± 1.9	**−0.49 (−0.94, −0.05)**

Abbreviations: MET, metformin; TKA, total knee arthroplasty; PR, prevalence ratio; SD, standard deviation; IPTW, inverse probability of treatment weighting; ^†^ without adjustment; ^‡^ adjusted for age, gender, body mass index, celecoxib, etoricoxib, tramadol, and insurance status; ^§^ age, gender, body mass index, celecoxib, etoricoxib, tramadol, and insurance status were incorporated into the propensity score model using inverse probability of treatment weighting. Those with statistical significance are shown in bold.

**Table 3:** Associations between metformin use in different durations and the risk of total knee arthroplasty.

MET Duration ^a^	Number	TKA, No. (%)	Univariable ^†^		Multivariable ^‡^		IPTW ^§^	
			PR (95% CI)	*p*	PR (95% CI)	*p*	PR (95%CI)	*p*
0	442	83 (18.8)	Reference		Reference		Reference	
1	210	14 (6.7)	**0.31 (0.16, 0.54)**	**<0.001**	**0.37 (0.18, 0.69)**	**0.003**	**0.44 (0.23, 0.84)**	**0.014**
2	210	7 (3.4)	**0.15 (0.06, 0.31)**	**<0.001**	**0.17 (0.07, 0.36)**	**<0.001**	**0.17 (0.07, 0.37)**	**<0.001**
*p* for trend				**<0.001**		**<0.001**		

Abbreviation: MET, metformin; TKA, total knee arthroplasty; PR, prevalence ratio; CI, confidence interval; IPTW, inverse probability of treatment weighting; ^a^ 0 represents no metformin, 1 represents short-term metformin use, and 2 represents long-term metformin use; ^†^ without adjustment; ^‡^ adjusted for age, gender, body mass index, celecoxib, etoricoxib, tramadol, and insurance status; ^§^ age, gender, body mass index, celecoxib, etoricoxib, tramadol, and insurance status were incorporated into the propensity score model using inverse probability of treatment weighting. Those with statistical significance are shown in bold.

**Table 4:** Associations between metformin use in different durations and degrees of knee pain.

MET Duration ^a^	Number	Knee Pain (Mean ± SE)	Univariable ^†^		Multivariable ^‡^		IPTW ^§^	
			β (95% CI)	*p*	β (95% CI)	*p*	β (95% CI)	*p*
0	155	2.1 ± 0.1	Reference		Reference		Reference	
1	94	1.6 ± 0.2	**−0.53 (−0.98, −0.09)**	**0.020**	**−0.51 (−1.00, −0.02)**	**0.042**	−0.39 (−0.91, 0.13)	0.141
2	47	1.6 ± 0.3	−0.55 (−1.12, 0.03)	0.065	−0.43 (−1.03, 0.17)	0.164	−0.50 (−1.14, 0.13)	0.124
*p* for trend				**0.017**		0.062		

Abbreviation: MET, metformin; SE, standard error; CI, confidence interval; IPTW, inverse probability of treatment weighting; ^a^ 0 represents no metformin, 1 represents short-term metformin use, and 2 represents long-term metformin use; ^†^ without adjustment; ^‡^ adjusted for age, gender, body mass index, celecoxib, etoricoxib, tramadol, and insurance status; ^§^ age, gender, body mass index, celecoxib, etoricoxib, tramadol, and insurance status were incorporated into the propensity score model using inverse probability of treatment weighting. Those with statistical significance are shown in bold.

## Data Availability

The data that support the findings of this study are available on the request from the corresponding author.

## Supplementary Materials

The following supporting information can be downloaded at: https://www.mdpi.com/article/10.3390/jcm11164796/s1, Figure S1: Distribution of propensity scores between MET and Non-MET subjects; Table S1: Associations between metformin use in different doses and risk of total knee arthroplasty; Table S2: Associations between metformin use in different doses and degrees of knee pain.
