# Supplementary material for: Association between Metformin Use and Risk of Total Knee Arthroplasty and Degree of Knee Pain in Knee Osteoarthritis Patients with Diabetes and/or Obesity: A Retrospective Study
Source: J Clin Med. 2022 Aug 17;11(16):4796. doi: 10.3390/jcm11164796 (PMC9409735; doi:10.3390/jcm11164796)
Supplement: Supplementary file 1 [file jcm-11-04796-s001.zip › jcm-1801275-supplementary.pdf]

Association between metformin use and risk of total knee arthroplasty and degree of knee pain in knee osteoarthritis patients with diabetes and/or obesity: a retrospective study

Supplementary tables

Supplementary Table S1 Associations between metformin use in different doses and risk of total knee arthroplasty

MET dose [a]	Number	TKA, No. (%)	Univariable†		Multivariable‡		IPTW§	
			PR (95%CI)	*p*	PR (95%CI)	*p*	PR (95%CI)	*p*
0	442	83 (18.8)	Reference		Reference		Reference	
1	210	13 (6.2)	**0.29 (0.15, 0.51)**	**0.004**	**0.34 (0.17, 0.65)**	**0.002**	**0.40 (0.20, 0.78)**	**0.008**
2	210	8 (3.8)	**0.17 (0.08, 0.34)**	**<0.001**	**0.19 (0.08, 0.41)**	**<0.001**	**0.21 (0.10, 0.44)**	**<0.001**
P for trend				**<0.001**		**<0.001**		

a 0 represents no metformin, 1 represents low metformin dose, 2 represent high metformin dose.

†Without adjustment.

‡Adjusted for age, gender, body mass index, celecoxib, etoricoxib, tramadol and insurance status.

§Age, gender, body mass index, celecoxib, etoricoxib, tramadol and insurance status were incorporated into the propensity score model using inverse probability of treatment weighting.

Those with statistical significance are shown in bold.

Abbreviation: MET, metformin; TKA, total knee arthroplasty; PR, prevalence ratio; CI, confidence interval; IPTW, inverse probability of treatment weighting.

Supplementary Table S2 Associations between metformin use in different doses and degree of knee pain

MET dose [a]	Number	Knee pain (mean ± SE)	Univariable†		Multivariable‡		IPTW§	
			β (95%CI)	*p*	β (95%CI)	*p*	β (95%CI)	*p*
0	173	2.1 ± 0.1	Reference		Reference		Reference	
1	94	1.5 ± 0.2	**-0.57 (-1.02, -0.12)**	**0.014**	**-0.56 (-1.05, -0.07)**	**0.025**	-0.42 (-0.94, 0.09)	0.108
2	47	1.6 ± 0.3	-0.48 (-1.06, 0.10)	0.103	-0.34 (-0.94, 0.26)	0.269	-0.46 (-1.10, 0.17)	0.151
P for trend				**0.024**		0.094		

a 0 represents no metformin, 1 represents low metformin dose, 2 represent high metformin dose.

†Without adjustment.

‡Adjusted for age, gender, body mass index, celecoxib, etoricoxib, tramadol and insurance status.

§Age, gender, body mass index, celecoxib, etoricoxib, tramadol and insurance status were incorporated into the propensity score model using inverse probability of treatment weighting.

Those with statistical significance are shown in bold.

Abbreviation: MET, metformin; PR, prevalence ratio; SE, standard error; CI, confidence interval; IPTW, inverse probability of treatment weighting.

Supplementary figures

Supplementary Figure S1 The distribution of propensity scores between metformin use and non-use groups

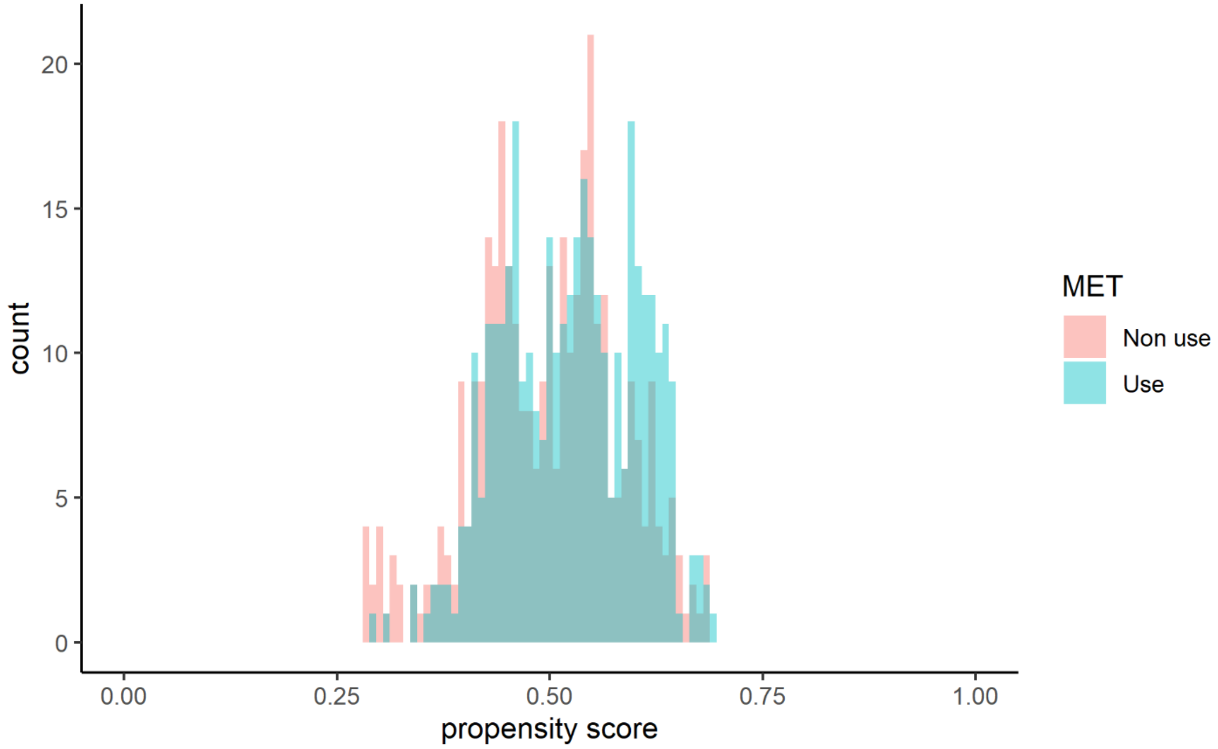

Abbreviation: MET, metformin
